# Pregnancy and Pregnancy Outcomes in Women with Eating Disorders: A Four-Year Longitudinal Study with Case Series

**DOI:** 10.3390/pediatric17060114

**Published:** 2025-11-03

**Authors:** Bárbara César Machado, Sónia Gonçalves, Sofia Duarte, Isabel Brandão, António Roma-Torres, Filipa Soares

**Affiliations:** 1 CEDH—Research Centre for Human Development, Faculdade de Educação e Psicologia, Universidade Católica Portuguesa, 4169-005 Porto, Portugal; filipamsoares@gmail.com; 2 Psychology School, University of Minho, 4704-553 Braga, Portugal; sgoncalves@psi.uminho.pt; 3 Psychiatry Department, Faculty of Medicine, Hospital of S. João, Alameda Prof. Hernâni Monteiro, 4200-319 Porto, Portugal; sofia.duarte.silva@sapo.pt (S.D.); isabelmbrandao@gmail.com (I.B.); aroma@sapo.pt (A.R.-T.)

**Keywords:** pregnancy, eating disorders, postpartum, course and outcome of ED symptoms, baby and children’s development

## Abstract

Background/Objectives: Eating disorders (EDs) often affect fertility, yet many women with ED still become mothers. The pattern of ED symptoms during pregnancy and postpartum, along with their effects on maternal and child health, is not yet fully understood. This longitudinal study aimed to (1) examine the course of ED symptoms from conception to postpartum, (2) evaluate pregnancy outcomes and children’s health and developmental milestones, and (3) assess ED status approximately four years after the initial evaluation. Methods: Thirty women with a prior ED diagnosis (21 with anorexia nervosa, 9 with bulimia nervosa) were evaluated at two time points. Time 1 with the Eating Disorders Examination and the Oxford Risk Factors for Eating Disorders: Interview Schedule; Time 2, approximately four years later, with the Eating Disorders Examination and the Clinical Interview on Reproductive History and Eating Behavior that also included clinical data related to mother’s health and baby’s health and development accessed through the Pregnant Women’s Health Bulletin and the Child and Youth Health Bulletin using the national health records. Results: ED symptoms (dietary restriction, self-induced vomiting, laxative misuse) persisted from conception through postpartum. BN participants reported more severe symptoms and higher rates of pregnancy complications (hyperemesis gravidarum, gestational diabetes, preeclampsia), while premature births occurred only in AN participants. Children of mothers with AN more frequently showed delays in developmental milestones (sitting, walking, speaking, sphincter control) compared to those of BN mothers. Conclusions: A substantial proportion of women with prior ED continued to experience symptoms during and after pregnancy, and nearly half still met diagnostic criteria four years later and are still in treatment. Cognitive features such as body dissatisfaction persisted despite partial symptom remission. These findings highlight the chronicity of ED and underscore the need for systematic screening, psychological support, and interdisciplinary follow-up during pregnancy and early motherhood.

## 1. Introduction

The age range for eating disorders (ED) overlaps with the age range for reproductive function, and for that reason, ED during pregnancy may occur in a significant number of women. A recent systematic review and meta-analysis [1] estimated a 4.3% prevalence of ED among pregnant women, highlighting ED as an important health issue during this period. Pregnancy involves profound physical and psychological changes, which may increase the risk of symptom exacerbation or the onset of ED-related concerns, particularly body image dissatisfaction. Recent studies also emphasize that ED during pregnancy is associated with a higher likelihood of maternal and neonatal complications, including obstetric difficulties, prematurity, low birth weight, and, in cases of binge eating disorder, macrosomia and delivery complications [2,3,4].

Regarding the course of ED symptoms during pregnancy, studies have reported heterogeneous trajectories. Bannatyne et al. [5] classified these into three categories: ED present before pregnancy, ED exacerbated during pregnancy, and ED with onset during pregnancy. While some studies [6,7] suggest a general symptom improvement or remission during pregnancy, others [8] point to increased vulnerability and symptom deterioration, particularly concerning binge eating and purging behaviors. The postpartum period has consistently been described as a vulnerable phase, marked by a higher risk of relapse or symptom worsening compared to pregnancy [8].

Independent of the course of the symptoms, the occurrence of ED symptoms during pregnancy is associated with adverse maternal and child outcomes. Beyond obstetric and neonatal risks, the postpartum phase presents additional challenges. Women with ED report greater breastfeeding difficulties and more frequent mealtime conflicts with their children, which have been linked to infant growth issues and strained mother–child interactions [9,10]. Moreover, research highlights potential long-term developmental consequences, including difficulties in attachment quality and emotional regulation, although evidence remains scarce and often based on small samples [8,11].

In sum, despite advances in the field, significant gaps remain. Most studies have been conducted in Northern Europe and North America, with data from Southern European countries being scarce. To our knowledge, there are no national data on ED during pregnancy in Portugal, and this is among the first longitudinal studies to examine this topic in the Portuguese context. Furthermore, few studies have explored ED longitudinally from conception through the postpartum period and followed up offspring development after birth. Addressing these gaps is essential for both clinical practice and public health, given the potential impact of ED on maternal and child health. Therefore, and considering a sample of women diagnosed with anorexia nervosa (AN) or bulimia nervosa (BN) before pregnancy, we designed a longitudinal study based on a case series with the following aims: (1) examine the presence and course of ED symptoms from conception and pregnancy to postpartum, (2) evaluate the pregnancy outcomes and children’s health and major developmental tasks, and (3) assess the course and outcome of ED symptoms and determining current ED status approximately four years after the first evaluation.

## 2. Method

### 2.1. Participants

The study included 30 women aged between 26 and 48 years (*M* = 34.80, *SD* = 5.59). Twenty-one women (70%) have been diagnosed with AN and nine women (30%) with BN before pregnancy and according to the criteria of the Diagnostic and Statistical Manual of Mental Disorders [12]. Thirteen participants (43%) were currently receiving treatment for ED in an ED-specialized unit. The sociodemographic characteristics of the participants are summarized in Table 1.

### 2.2. Measures

#### 2.2.1. Eating Disorders Examination (EDE) 

The EDE [13] is a semi-structured, researcher-based interview designed to assess the specific psychopathological features of ED, including symptom presence and frequency, as well as extreme concerns related to body shape and weight [14]. It provides detailed descriptive information on ED symptoms and diagnoses [15]. The EDE is considered the gold standard for the assessment of ED psychopathology, with extensive use in both clinical and research settings. It demonstrates excellent inter-rater reliability and internal consistency [15,16,17], including in women during the perinatal period, supporting its validity in samples comparable to ours.

#### 2.2.2. Oxford Risk Factors for Eating Disorders: Interview Schedule (RFI) 

Section 2.7 of the RFI [18]—Reproductive History—was used to collect detailed information on pregnancy in women with ED. The RFI is a semi-structured, researcher-based interview focused on risk factors associated with AN and BN, developed from a biopsychosocial perspective [19]. RFI section 2.7 covers three domains: (i) reproductive vulnerability and history (number of children, number of pregnancies, presence of spontaneous or induced abortions, stillbirths, detection of congenital anomalies, health problems during pregnancy, dieting for weight control, compensatory behaviors, and eating habits); (ii) difficulties with conception (duration of attempts, dieting to influence weight and body shape); and (iii) disordered eating. The RFI provides a standardized framework for collecting retrospective reproductive data in women with ED, minimizing recall bias using operationalized behavioral definitions. Previous studies have demonstrated good inter-rater reliability and validity of the instrument in assessing reproductive and developmental risk factors in women with AN and BN [19].

#### 2.2.3. Clinical Interview on Reproductive History and Eating Behavior (ECHRCA) 

A separate semi-structured interview, the ECHRCA [20], was conducted to complement information on preconception and pregnancy periods for women with ED (obtained from Section 2.7 of the RFI). It covers five domains of reproductive history: (i) Gestational Development, (ii) Neonatal Period (0–25 days), (iii) Postpartum, (iv) Clinical Issues, and (v) Medical History of the Child. The ECHRCA was specifically developed by our team to address reproductive history and early child outcomes in mothers with ED, providing cultural and contextual adequacy to the present study. We checked for agreement with the medical records whenever necessary, reinforcing its utility as a complementary tool for internationally established interviews.

#### 2.2.4. Pregnant Women’s Health Bulletin and the Child and Youth Health Bulletin 

Both medical forms from the Portuguese National Health System records [21,22] were filled in by the Medical Doctors (Obstetrician and Pediatrician) who followed the mothers and the babies. Data about pregnancy, birth, and post-birth phases, and newborn and children’s development were based on these records. Importantly, information on the achievement of major developmental milestones (e.g., sitting, walking, speaking, sphincter control) was obtained directly from the Child and Youth Health Bulletin, which is systematically updated by pediatricians during child visits in the first 36 months of life. Children’s development major tasks were therefore evaluated during the first 36 months using these standardized medical records.

### 2.3. Procedure

Participants were recruited and assessed at two time points at a specialized ED treatment center. At Time 1, participants with AN or BN who had experienced at least one previous successful pregnancy were interviewed using the EDE diagnostic items [13] to assess and confirm ED symptoms and diagnosis accurately. The RFI [18] was also administered to evaluate reproductive history and the course of ED symptomatology from conception through pregnancy and the postpartum period. Approximately four years later, at Time 2, participants were re-contacted to schedule follow-up interviews aimed at assessing the course and outcome of ED symptoms and determining current ED status. The same EDE interview [13] and the ECHRCA [20] were used to assess the child’s health and achievement of key developmental milestones. Additional clinical data regarding maternal and child health and development were gathered from the Pregnant Women’s Health Bulletin and the Child and Youth Health Bulletin [21,22]. Interviews were conducted face-to-face at the central hospital ED treatment unit by clinical psychologists trained in using the standardized administration of the EDE and RFI.

Inclusion criteria were a diagnosis of an ED before conception and at least one successful pregnancy. Exclusion criteria included the presence of physical illnesses likely to affect eating behaviors or weight, and psychotic disorders. Ethical standards were upheld, with informed consent obtained from all participants and ethical approval granted by the institution’s Ethics Committee, as the study is part of a broader research project on eating disorders.

The research was conducted in accordance with the principles outlined in the Declaration of Helsinki [23] and approved by the Ethics Committee of Centro Hospitalar São João. The consent form for participation was distributed to all participants and signed.

### 2.4. Data Analysis

Statistical analyses were performed using IBM SPSS Statistics (version 30.0) [24]. Descriptive statistics (means, standard deviations, ranges, and percentages) were used to characterize sociodemographic, clinical, and obstetric variables, as well as newborn outcomes. To examine potential group differences (AN vs. BN) in continuous variables such as newborn birth weight, newborn length, and breastfeeding duration, non-parametric Mann–Whitney *U* tests were conducted, given the small sample size and non-normal distribution of data. Categorical variables (e.g., obstetric complications, remission rates) were compared descriptively, using absolute and relative frequencies. The statistical significance threshold was set at *p* < 0.05 (two-tailed).

## 3. Results

### 3.1. Time 1

#### 3.1.1. Conception Period

Before pregnancy, 12 women (40%) reported being on a restrictive diet (6, 28.6% AN group; 6, 66.7% BN group), 5 (16.7%) using self-induced vomit (1, 4.8% AN group; 4, 44.4% BN group) and 2 (6.7%) the use of laxatives (0, AN group; 2, 22.2% BN group). BMI ranged between 14.83 and 25.59 (*M* = 19.85; *SD* = 2.96) for the AN group and 18.83 and 22.86 (*M* = 20.37; *SD* = 1.23) for the BN group.

One-third of the women (*n* = 10) reported difficulties in getting pregnant (5, 23.8% AN group; 5, 55.6% BN group), 11 (36.7%) reported medical problems before pregnancy (i.e., polycystic ovaries, oligomenorrhea and amenorrhea; 8, 38.1% AN group; 3, 33.3% BN group), and 7 (23.3%) reported having a previous abortion (4, 19% AN group; 3, 33.3% BN group). Table 2 presents the results for the conception period.

#### 3.1.2. Pregnancy Period

During pregnancy, 17 women (58%) reported the feeling of being fat (11, 52.4% AN group; 6, 66.6% BN group), 16 (52%) being on a diet (9, 42.8% AN group; 7, 77.8% BN group), 8 (26.6%) using self-induced vomit (3, 14.3% AN group; 5, 55.5% BN group) and 2 (6.7%) the use of laxatives (1, 4.8% AN group; 1, 11.1% BN group). Mothers’ BMI at the end of the pregnancy ranged between 17.99 and 32.05 (*M* = 24; *SD* = 4.08) for the AN group and 23.01 and 30.84 (*M* = 26.11; *SD* = 2.53) for the BN group.

Fourteen women (46.7%) were diagnosed with a high-risk pregnancy by their obstetricians (e.g., premature placental abruption, premature aging of the placenta, twin pregnancy; 10, 47.6% AN group; 4, 44.4% BN group) and 12 (66.7%) reported clinical problems during pregnancy like hyperemesis gravidarum, gestational diabetes, and preeclampsia (6, 28.6% AN group; 6, 66.7% BN group). Pregnancy period results are summarized in Table 3.

#### 3.1.3. Delivery and the Postpartum Period

Regarding delivery, 14 (46.7%) women reported having a cesarean (11, 52.4% AN group; 3, 33.3% BN group) and 8 (26.6%) having delivery with maneuvers (i.e., the use of forceps and/or suction cups; 5, 23.8% AN group; 3, 33.3% BN group). Pregnancy length ranged between 28 and 42 weeks for the AN group (*M* = 37.52; *SD* = 3.20) and between 37 and 40 weeks for the BN group (*M* = 38.78; *SD* = 0.97); 6 (20%) participants reported having a premature child (6, 28.6% AN group; 0, BN group). Most children were males (18, 60%; 13, 61.9% AN group; 5, 55.6% BN group). Twenty-five women (83.3%; 17, 81% AN group; 8, 88.9% BN group) reported breastfeeding their children lasting for 1 to 36 months (*M* = 10.24, *SD* = 10.61). No significant differences were found in breastfeeding duration between mothers with AN and mothers with BN (*U* = 66.000, *p* = 0.907). Twenty-six (86.7%) of the newborns were at normal weight (≥2601 g ≤4000 g; 17, 80.9% from mothers of the AN group; 6, 66.6% from mothers of the BN group) and 26 (86.6%) were at standard length (≥46.0 cm ≤54.0 cm; 17, 81% from mothers of the AN group; 9, 100% from mothers of the BN group). No significant differences in baby weight and length were found between mothers with AN and mothers with BN (*U* = 82.500, *p* = 0.577; *U* = 60.500, *p* = 0.111).

During postpartum, 8 (26.7%) women reported the use of self-induced vomiting (3, 14.3% AN group; 5, 55.6% BN group), and 15 (50%) restrictive diet, and/or self-induced vomiting, and/or laxative misuse (9, 42.9% AN group; 6, 66.7% BN group). Four weeks after delivery, BMI ranged between 14.87 and 28.65 (*M* = 21.41; *SD* = 3.83) for the AN group and 15.06 and 30.04 (*M* = 23.30; *SD* = 4.56) for the BN group. Table 4 summarizes delivery and postpartum results.

### 3.2. Time 2

#### 3.2.1. ED Symptoms and Diagnosis

After approximately four years of the first assessment, 16 (53.3%) women presented AN or BN complete remission (14, 66.7% AN group; 2, 22.2% BN group), 9 (30%) were in partial remission (6, 28.6% AN group; 3, 33.33% BN group), and 5 (16.7%) maintained AN or BN complete diagnosis (1, 4.8% AN group; 4, 44.4% BN group). All the participants who maintained AN symptoms were undergoing treatment. For participants who maintain BN symptoms, only one participant was not undergoing treatment.

In the previous month of the Time 2 assessment, 16 (53.3%) women reported being on a diet (10, 47.6% AN group; 6, 66.6% BN group), 8 (26.7%) the presence of binge eating (3, 14.3% AN group; 5, 55.5% BN group), 7 (23.3%) the use of self-induced vomit (1, 4.8% AN group; 6, 66.7% BN group), 2 (6.6%) laxative misuse (0, AN group; 2, 22.2% BN group), 1 (3.3%) diuretic misuse (0, AN group; 1, 11.1% BN group), and 4 (13.3%) excessive/compulsive exercise (2, 9.6% AN group; 2, 22.2% BN group). Four (13.3%) participants reported amenorrhea or menstrual irregularities (3, 14.3% AN group; 1, 11.1% BN group). BMI ranged between 12.37 and 25.59 (*M* = 20.08; *SD* = 3.47) in the AN group and 18.14 and 26.56 (*M* = 21.62; *SD* = 2.53) in the BN group. ED symptoms and diagnosis results, along with other specific psychopathological features of ED, are summarized in Table 5. For both groups, we found the presence of ED symptoms to be superior to the number of women who maintain the ED diagnosis in the current assessment. As we can see, most of the women from the BN group maintain extreme concerns related to body shape and weight. Despite the same pattern for women of the AN group, women of the BN group reported subjective bulimic episodes, and higher results related to feeling fat, desire to lose weight, importance of weight, negative self-evaluation due to shape/weight, fear of weight gain, discomfort seeing body, discomfort about exposure, discomfort in social or public situations because of shape, and vigilance about shape.

#### 3.2.2. Babies’ and Children’s Health and Major Development Tasks

Concerning the baby’s health, 5 (16.7%) were diagnosed with health problems during the first month (i.e., cardiac arrest, bronchopulmonary dysplasia, septicemia, gastric reflux, ocular problems; 3, 14.3% AN group; 2, 22.2% BN group). None were diagnosed with congenital abnormalities. Regarding the baby’s and children’s development, the age of the significant development tasks evaluated was, as mentioned above, accessed in the Portuguese Child and Youth Health Bulletin reports. Additionally, these tasks’ average and no average ages were evaluated considering this document’s guidelines. Accordingly, 5 (16.7%) babies sit down without help later than average (5, 23.8% AN group; 0, BN group), 5 (16.7%) walk alone later than average (5, 23.8% AN group; 0, BN group), 9 (30%) babies started to speak later than average (8, 38.1% AN group; 1, 11.1% BN group) and 2 (6.7%) had sphincter control later than average (2, 9.5% AN group; 0, BN group). Table 6 summarizes babies and children’s health and major developmental tasks results.

## 4. Discussion

This longitudinal study aimed to evaluate 30 women diagnosed with ED before pregnancy in a time frame of two moments: Time 1, focused on conception, pregnancy, and postpartum, and Time 2, assessing pregnancy outcomes, children’s health, major developmental milestones, and ED symptoms.

In line with the study objectives, the discussion is structured around three domains: (1) trajectory of ED symptoms across conception, pregnancy, and postpartum, (2) obstetric and child developmental outcomes, and (3) the diagnostic course at four years.

### 4.1. Symptom Trajectory

During the conception phase, the most common symptoms were diet and self-induced vomiting, both of which were greater in the BN group. A considerable proportion of the women also reported difficulties conceiving. While causality cannot be inferred, this finding suggests a possible association between ED and fertility difficulties, consistent with previous reports [25]. Additionally, almost one-quarter reported having a previous abortion. This pattern may reflect an increased vulnerability of women with ED to adverse reproductive health outcomes.

Throughout pregnancy, most of the women reported the feeling of being fat, the presence of a restrictive diet, and self-induced vomiting. Koubaa et al. [26] have emphasized the psychological impact of weight concerns, which may intensify with the physical changes in pregnancy. Soest and Wichstrøm [27] similarly found that weight concern and body dissatisfaction are closely tied to eating problems during motherhood. In our sample, self-induced vomiting persisted in both AN and BN participants, with higher rates in BN, suggesting symptomatic maintenance rather than remission. This contrasts with previous reports of symptom improvement during pregnancy [28,29].

### 4.2. Obstetric and Child Outcomes

We also found higher general ED symptoms and health issues in BN participants and premature births occurring only among AN participants. Specifically, for AN, the literature points to premature birth and low birth weight, potentially related to low maternal BMI [30]. In the general population, the prevalence of preterm births is estimated at 5–7% in high-income countries [31], suggesting that the rate observed in our AN subgroup may represent an elevation in risk. Other studies also found adverse neonatal outcomes, such as preterm birth, particularly associated with AN [25,30].

In our study, a significant number of women were diagnosed with a high-risk pregnancy by their obstetricians (e.g., premature placental abruption, premature aging of the placenta), and most of the women from the BN group presented clinical problems during pregnancy (e.g., hyperemesis gravidarum, gestational diabetes, preeclampsia). Other studies [32] have also identified increased health complications during pregnancy and more frequent obstetric problems.

Regarding obstetric maneuvers, most participants reported a high rate of cesarean sections or forceps-assisted deliveries in both AN and BN groups. In the general population, cesarean rates in Europe and North America range between 25 and 35% [33], which is lower than the rates observed in our sample, suggesting that women with ED may undergo surgical deliveries more frequently.

Concerning obstetric complications and infant health, our findings suggest that almost all newborns were at normal weight and length. This may reflect the fact that most AN participants had improved their BMI range at the end of pregnancy, potentially mitigating infant birth weight effects [34].

Regarding child development, most children achieved developmental tasks within expected ranges. However, some delays (e.g., sitting, walking, speaking, sphincter control) were more frequent among children of AN mothers. Given that approximately 5–10% of children in the general population experience developmental delays [35], the rates observed in our AN subgroup suggest an increased risk, albeit based on small numbers.

### 4.3. Diagnostic Course at Four Years

Approximately four years after the first assessment, almost half of the participants evaluated maintained the ED diagnosis and remained in treatment in ED specialized settings. The remission rate was higher for AN than for BN. This finding underlines the chronicity of BN symptoms and is consistent with long-term follow-up studies showing poorer prognosis in BN [36]. Importantly, even among those without a current ED diagnosis, many participants reported ongoing cognitive features such as body dissatisfaction and weight/shape overvaluation. These results highlight the need for continued monitoring beyond the perinatal period, as subclinical symptoms can perpetuate functional impairment and risk of relapse.

### 4.4. Clinical Implications

The observed outcomes may be explained by multiple interacting mechanisms: psychological factors (e.g., body image concerns, heightened anxiety during pregnancy), nutritional aspects (low BMI, inadequate gestational weight gain), and social determinants (limited partner/family support, stigma). These mechanisms can adversely influence both maternal and child health.

From a clinical perspective, our findings underscore the importance of: (1) systematic screening for ED symptoms in prenatal care, (2) psychoeducation for pregnant women with ED about risks and protective factors regarding ED symptoms, (3) specialized psychological interventions targeting body image and emotion regulation, and (4) coordinated postpartum follow-up, including pediatric monitoring of child development. These recommendations align with recent clinical guidelines for the perinatal period [37].

## 5. Conclusions

This study showed that ED symptoms (dietary restriction, self-induced vomiting, laxative misuse) frequently persisted from conception through postpartum. Four years later, almost half of the women continued to meet diagnostic criteria, with many others reporting enduring cognitive symptoms (e.g., body dissatisfaction, overvaluation of weight/shape). Consistent with previous evidence [38], these findings highlight the long-term psychological and social consequences of ED, even beyond weight normalization. Compared to AN, BN was associated with a less favorable course and lower remission rates.

Contrary to studies suggesting symptomatic improvement during pregnancy [32,38], our findings indicate persistence of symptoms, particularly in BN. Pregnancy appeared to be more destabilizing for BN than for AN, whereas remission rates were relatively higher in AN participants [39]. The observation of increased health problems in BN pregnancies and preterm births in AN underscores the need for close obstetric monitoring of women with a history of ED, in line with prior recommendations [40].

Taken together, these results suggest that pregnancy does not necessarily attenuate ED symptoms and that a considerable proportion of women continue to struggle years later, reinforcing the chronic nature of ED and the need for sustained intervention [38].

The current study has several limitations. The most important one is associated with the limited number of participants enrolled in the study. Another limitation is due to retrospective designs, namely, potential biases associated with recall. We made every effort to maximize recall accuracy, but bias is unavoidable. Therefore, we also considered the records from the Medical Doctors who followed the mothers and the babies. We also tried to overcome these limitations by having all the interviews performed by clinical psychologists with current clinical practice in evaluating and intervening with ED.

Despite these limitations, this study modestly contributes to understanding the relationship between reproductive history and ED. Further research with a larger prospective cohort is needed to clarify prognosis and mechanisms related to different pathways. Clinically, our results reinforce the recommendations from recent perinatal guidelines [37]: (1) early detection and systematic screening of ED during pregnancy, (2) psychoeducation and support for the mother, (3) cross-system collaboration between obstetrics, pediatrics, nutrition, and mental health services, (4) tailored psychological and medical treatment, and (5) continued monitoring into the postpartum years. These measures are essential to minimize risks for both mother and child.

## Figures and Tables

**Table 1:** Sociodemographic characterization of the two clinical groups (AN and BN).

	AN (*n* = 21)		BN (*n* = 9)	
Age	Range	*M* (*SD*)	Range	*M* (*SD*)
	28–48	35.76 (5.69)	26–40	32.56 (4.90)
Number of children	1–2	1.38 (0.50)	1–2	1.33 (0.50)
First child age (months)	16–215	87.05 (63.97)	34–195	107.0 (53.51)
First child gender	*n* (%)		*n* (%)	
Male	13 (61.9)		5 (55.6)	
Female	8 (38.1)		4 (44.4)	
Marital status				
Single	2 (9.5)		0 (0)	
Married	17 (81.0)		7 (77.8)	
Non-marital partnership	–2 (9.5)		1 (11.1)	
Divorced	0 (0)		1 (11.1)	
Professional status				
Employed	13 (61.9)		7 (77.8)	
Unemployed	7 (33.3)		2 (22.2)	
Disability retirement	1 (4.80)		0 (0)	
Educational grade				
Primary School 1st Cycle	1 (4.8)		0 (0)	
Primary School 2nd Cycle	1 (4.8)		0 (0)	
Primary School 3rd Cycle	4 (19.0)		4 (44.4)	
Secondary school attendance	1 (4.80)		0 (0)	
Completed secondary school	4 (19.0)		2 (22.2)	
Completed higher education	10 (47.6)		3 (33.3)	

*M*—mean; *SD*—standard deviation.

**Table 2:** Conception period of the two clinical groups (AN and BN).

	AN (*n* = 21)	BN (*n* = 9)	*N* = 30
Diet	6 (28.6%)	**6 (66.7%)**	12 (40.0%)
Self-induced vomiting	1 (4.8%)	**4 (44.4%)**	5 (16.7%)
Laxative misuse	0 (0.0%)	**2 (22.2%)**	2 (6.7%)
BMI range BMI (*M*, *SD*)	14.83–25.59 19.85 (2.96)	18.83–22.86 20.37 (1.23)	–
Difficulties getting pregnant	5 (23.8%)	**5 (55.6%)**	10 (33.3%)
Medical problems before pregnancy *	**8 (38.1%)**	3 (33.3%)	11 (36.7%)
Previous abortion	4 (19.0%)	**3 (33.3%)**	7 (23.3%)

*M*—mean; *SD*—standard deviation. * Medical problems include polycystic ovaries, oligomenorrhea, and amenorrhea.

**Table 3:** Pregnancy period of the two clinical groups (AN and BN).

	AN (*n* = 21)	BN (*n* = 9)	*N* = 30
Feeling fat	11 (52.4%)	**6 (66.6%)**	17 (58%)
Diet	9 (42.8%)	**7 (77.8%)**	16 (52%)
Self-induced vomiting	3 (14.3%)	**5 (55.5%)**	8 (26.6%)
Laxative misuse	1 (4.8%)	1 (11.1%)	2 (6.7%)
BMI at end of pregnancy (range) BMI at end of pregnancy (*M*, *SD*)	17.99–32.05 24.0 (4.08)	23.01–30.84 26.11 (2.53)	–
High-risk pregnancy	**10 (47.6%)**	4 (44.4%)	14 (46.7%)
Clinical problems during pregnancy *	6 (28.6%)	**6 (66.7%)**	12 (40.0%)

*M*—mean; *SD*—standard deviation. * Clinical problems include hyperemesis gravidarum, gestational diabetes, and preeclampsia.

**Table 4:** Delivery and postpartum characteristics of the two clinical groups (AN and BN).

	AN (*n* = 21)	BN (*n* = 9)	*N* = 30
Cesarean delivery	**11 (52.4%)**	3 (33.3%)	14 (46.7%)
Delivery with maneuvers *	5 (23.8%)	**3 (33.3%)**	8 (26.6%)
Pregnancy length range Pregnancy length (*M*, *SD*)	28–42 37.52 (3.20)	37–40 38.78 (0.97)	–
Premature child	**6 (28.6%)**	0 (0.0%)	6 (20.0%)
Child’s sex (male)	13 (61.9%)	5 (55.6%)	18 (60.0%)
Breastfeeding	17 (80.9%)	8 (88.9%)	25 (83.3%)
Breastfeeding months duration range Breastfeeding months duration (*M*, *SD*)	1–36 10.24 (10.61)	1–36 10.24 (10.61)	–
Newborn weight			
<2600 g	4 (19.0%)	0	4 (13.3%)
2601–2900 g	6 (28.6%)	3 (33.3%)	9 (30%)
2901–3200 g	5 (23.8%)	3 (33.3%)	8 (26.7%)
3201–3600 g	2 (9.5%)	3 (33.3%)	5 (16.7%)
3601–4000 g	4 (19.0%)	0	4 (13.3%)
4001–4300 g	0	0	0
4301–4500 g	0	0	0
Newborn length			
<45.9 cm	4 (19%)	0	4 (13.3%)
46.0–47.0 cm	5 (23.8%)	2 (22.2%)	7 (23.3%)
47.1–49.0 cm	8 (38.1%)	3 (33.3%)	11 (36.7%)
49.1–50.0 cm	2 (9.5%)	1 (11.1%)	3 (10.0%)
50.1–53.0 cm	1 (4.8%)	3 (33.3%)	4 (13.3%)
53.1–54.0 cm	1 (4.8%)	0	1 (3.3%)
54.1–55.0 cm	0	0	0
Postpartum ED symptoms			
self-induced vomit	3 (14.3%)	**5 (55.6%)**	8 (26.7%)
restrictive diet, and/or self-induced vomiting, and/or laxative misuse	9 (42.9%)	**6 (66.7%)**	15 (50%)
BMI four weeks after delivery range BMI four weeks after delivery (*M*, *SD*)	14.87–28.65 21.41 (3.83)	15.06–30.04 23.30 (4.56)	–

*M*—mean; *SD*—standard deviation. * Use of forceps and/or suction cups.

**Table 5:** ED symptoms and diagnosis results, and other specific psychopathological features of ED in the two clinical groups (AN and BN).

	AN (*n* = 21)	BN (*n* = 9)	*N* = 30
Complete remission	**14 (66.7%)**	2 (22.2%)	16 (53.3%)
Partial remission	6 (28.6%)	**3 (33.3%)**	9 (30.0%)
Maintained diagnosis	1 (4.8%)	**4 (44.4%)**	5 (16.7%)
Treatment among maintained diagnosis	**1 (100%)**	3 (75.0%)	4 (80.0%)
Diet	10 (47.6%)	**6 (66.7%)**	16 (53.3%)
Binge eating	3 (14.3%)	**5 (55.6%)**	8 (26.7%)
Self-induced vomiting	1 (4.8%)	**6 (66.7%)**	7 (23.3%)
Laxative misuse	0 (0.0%)	**2 (22.2%)**	2 (6.7%)
Diuretic misuse	0 (0.0%)	**1 (11.1%)**	1 (3.3%)
Excessive/compulsive exercise	2 (9.5%)	**2 (22.2%)**	4 (13.3%)
Amenorrhea/menstrual irregularities	**3 (14.3%)**	1 (11.1%)	4 (13.3%)
BMI at Time 2 range BMI at TIME 2 (*M*, *SD*)	12.37–25.59 20.08 (3.47)	18.14–26.56 21.62 (2.53)	–
EDE [13]			
Subjective bulimic episodes	0	**5 (55.5%)**	5 (16.7%)
Feeling fat	8 (38.1%)	**7 (77.8%)**	15 (50%)
Weight dissatisfaction	12 (57.1%)	**8 (88.9%)**	20 (66.7%)
Desire to lose weight	10 (46.7%)	**8 (88.9%)**	18 (60%)
Shape dissatisfaction	12 (57.1%)	**7 (77.8%)**	19 (63.3%)
Importance of weight	10 (46.7%)	**8 (88.9%)**	18 (60%)
Importance of shape	11 (52.4%)	**8 (88.9%)**	19 (63.3%)
Negative self-evaluation due to shape/weight	9 (42.9%)	**6 (66.7%)**	15 (50%)
No acceptance of shape/weight	11 (52.4%)	**7 (77.8%)**	12 (60%)
Fear of weight gain	8 (38.1%)	**7 (77.8%)**	15 (50%)
Discomfort seeing the body	10 (46.7%)	**5 (55.6%)**	15 (50%)
Discomfort about exposure	9 (42.9%)	**5 (55.6%)**	14 (46.7%)
Social discomfort	8 (38.1%)	**6 (66.7%)**	14 (46.7%)
Vigilance about shape	9 (42.9%)	**7 (77.8%)**	16 (53.3%)

*M*—mean; *SD*—standard deviation.

**Table 6:** Baby’s and children’s health and major development tasks results of the two clinical groups (AN and BN).

	AN (*n* = 21)	BN (*n* = 9)	*N* = 30
Health problems during the first month *	3 (14.3%)	**2 (22.2%)**	5 (16.7%)
Children’s development			
Sit down later than average	**5 (23.8%)**	0	5 (16.7%)
Walk later than average	**5 (23.8%)**	0	5 (16.7%)
Speak later than average	**8 (38.1%)**	1 (11.1%)	9 (30%)
Sphincter control is later than average	**2 (9.5%)**	0	2 (6.7%)

* Health problems include cardiac arrest, bronchopulmonary dysplasia, septicemia, gastric reflux, and ocular problems.

## Data Availability

The original contributions presented in this study are included in the article. Further inquiries can be directed to the corresponding author.
